# Ladarixin Potential over the Effects of IL-8 and of Serum from Patients with Abdominal Aortic Aneurysm on Human Aortic Cells

**DOI:** 10.3390/cells14211713

**Published:** 2025-10-31

**Authors:** Lucia Spartano, Maria Lombardi, Vincenzo Ardita, Roberto Chiesa, Andrea Aramini, Marcello Allegretti, Domenico Baccellieri, Lidia De Filippis, Chiara Foglieni

**Affiliations:** 1 Cardiovascular Research Center, IRCCS Ospedale San Raffaele, 20132 Milan, Italy; 2 Division of Vascular Surgery, IRCCS Ospedale San Raffaele, Vita-Salute San Raffaele University, 20132 Milan, Italy; 3 Dompé Farmaceutici S.p.A., 20122 Milan, Italy

**Keywords:** primary human aortic endothelial (HAOEC), primary smooth muscle cells (HAOSMC), Interleukin 8 (IL-8), CXCR2/CXCL1/IL-8 axis, Metalloprotease 9 (MMP9), Ladarixin (Lad)

## Abstract

Early cellular alterations in abdominal aortic aneurysm (AAA) are scarcely investigated. Aortic remodeling inflammation-related suggested the CXCR2/CXCL1/IL-8 axis as a therapeutic target. This study investigates CXCR1/CXCR2 antagonism in primary human aortic endothelial (HAOEC) and smooth muscle cells (HAOSMC) conditioned with IL-8 or serum from patients with AAA (sPT). Ladarixin (10 μM Lad or 25 μM) served as an inhibitor. Readouts included RT-qPCR for *CXCL1*, *CXCL8*, *CXCR2*, *MMP9*, *NFKB1*, and *VEGF-A*; zymography for MMP9 activity confocal microscopy for F-actin and mitochondria; NADPH/NADH diaphorase histochemistry for redox activity; and ATP assay. In HAOEC, IL-8 downregulated *CXCR2*, increased MMP9 activity, and induced cytoskeletal and mitochondria disorganization without altering NADH/NADPH diaphorases but increasing ATP release. At concentration of 10 μM Lad rescued cell organization and gene expression. sPT upregulated *CXCL8*, *CXCR2*, and *MMP9*, decreased NADH/NADPH diaphorases, and altered cytoskeleton and mitochondria organization in HAOEC. At concentration of 10 μM Lad (partially) and 25 μM Lad reverted gene upregulation and mitochondria distribution; both doses increased diaphorase and released ATP. HAOSMC were scantily susceptible to IL-8 and weakly responsive to sPT, slightly upregulating *CXCR2* and *VEGF-A* but increasing proMMP9 gelatinolysis. Ladarixin recovered proMMP9 activity and modulated *CXCL1*. AAA-like vascular cell alterations involve multiple inflammatory factors and are modulable by inhibition of IL-8 receptors. The results underline careful dose calibration.

## 1. Introduction

Vascular wall damage in abdominal aortic aneurism (AAA) involves complex cellular and molecular immune–inflammatory pathogenic factors [1]. Besides the amplificatory effect associated with intraluminal NETosis or thrombosis [2], the circulating inflammatory microenvironment and oxidative stress-associated deregulation of NAD(P)H enzymes and metalloproteases (MMPs) [3] may affect constitutive vascular wall cells from the early phases of AAA development. Endothelial changes precede the injury of underneath smooth muscle cells (SMC) and the loosening of the extracellular matrix [3]. The role of endothelial interaction with other cells belonging to the vascular wall during AAA formation has been postulated, but functional data about pro-aneurysmatic activation of the aortic cells are still scanty [3]. In LDR-/- mice, the deficiency of Angiotensin II receptor in aortic endothelium, but not in smooth muscle cells, attenuated the AAA development, and in the rat xenograft model of AAA involving dysendothelialization, rescue of the luminal lining by endothelial cell transplantation supported aortic wall stabilization through partially preserving the integrity of the internal elastic media [4,5]. The altered functionality of nitric oxide synthase and NAD(P)H oxidases in the endothelium activated by inflammation lead to increased production of oxygen radicals, inducing MMPs activation and abnormal matrix degradation, and affecting SMC functions, and ultimately contributing to aortic wall swelling and AAA development [3].

The administration of medications protecting the aortic luminal endothelium could be supportive in the reduction of the risk of AAA formation and rupture. This is relevant in the setting of AAA, as current pharmacological approaches (e.g., antihypertensive and antiplatelet drugs, statins, ACE inhibitors, and renin–angiotensin inhibitors) do not target AAA development but co-morbidities and risk factors. The identification of an optimal drug treatment is compulsory for going beyond monitoring of small AAA and surgery big AAA [1].

In the inflammatory background of AAA and specifically in the cyto-chemokines-related pathways, the CXCR2/CXCL1/IL-8 axis represents a putative therapeutic target in AAA, playing a causal role and pharmacologically modulable on *in vivo* murine elastase-induced AAA [6] and in *ex-vivo* cultured AAA tissues, as recently proposed by our group [7]. Here, we have mimicked the early phases of AAA in terms of activation of *in vitro* human aortic endothelial cells (EC) and SMC, exploring the dysfunctional effects of chronic stimulation with IL-8 or multiple circulating mediators on the homeostasis of the vascular wall cells.

We have also extended to activated EC and SMC the mechanistic investigation of the inhibition CXCR2/CXCL1/IL-8 axis, using the same dual CXCR1/CXCR2 antagonist Ladarixin (Lad) applied in the previous study [7]. Lad was mostly known for its application in cancer and type 1 diabetes, where this molecule is undergoing clinical trials [8]. Moreover, Lad contributed to protection against diabetic retinopathy in streptozotocin-treated rats [9] and impaired neutrophil migration without altering adhesive properties of the inflamed endothelium [10], hinting to a potential application in the vascular context. Accordingly with previous reports, including our recent findings on *ex vivo* cultured AAA tissues, we mainly focused on few readouts, i.e., *CXCR2/CXCL1/IL-8* and *MMP9* expressed genes, the activities of MMP9 and NAD(P)H oxidases, ATP release, mitochondrial and cytoskeletal organization [11,12,13,14].

## 2. Materials and Methods

### 2.1. Blood for Cell Conditioning

Serum samples (10 mL) from characterized male patients (Supplementary Table S2) with diagnosis of unruptured large AAA, according to the current European guidelines [15], hospitalized and undergoing infrarenal abdominal aortic aneurysmectomy at Vascular Surgery Unit of IRCCS San Raffaele Hospital, were used for cell conditioning. The donors were involved in a previously published study where further characteristics were described [14]. The research protocol (ID. AAA1, approved on 28 October 2021 and emended on 15 February 2023 by the Institutional Ethic Committee) conformed to the World Medical Association Declaration of Helsinki, and all enrolled individuals donating blood signed an informed consent.

Upon collection in vacutainer serum tubes, the blood was centrifuged at 2500× *g* 10 min at 4 °C, to obtain serum, which was aliquoted and stored at −80 °C until use.

The serum levels of several pro/anti-inflammatory mediators related to the CXCR2/CXCL1/IL-8 axis, namely CXCL1 CXCL2, IL-1α, IL-1β, IL-10, IL-6, IL-8, and TNF-α, were measured by Luminex multiplexed technology using FLEX MAP 3D LUMINEX instrument equipped with Bio Plex 6.2 software (all from R&D System, Minneapolis, MN, USA). Samples were stored at −80 °C and thawed only once, run in duplicate, and determined vs. standard curve, following the manufacturer’ instructions.

### 2.2. Cell Culture

Primary human aortic endothelial and smooth muscle cells (HAOEC and HAOSMC, from single donors 43 and 53 years old, Caucasian males, Cat. # 304-05a and # 354-05a, respectively) were cultured using specific cell culture medium (095211F-500 Human EC Growth Medium and 095311-500 Human SMC Growth Medium, respectively) following the manufacturer’ instructions and kept in standard 5% CO_2_ incubator at 37 °C. The materials were provided by Cell Applications, Inc., San Diego, CA, USA.

Two chronic conditioning stimuli were studied: human recombinant IL-8 (human recombinant IL-8, SRP3098, Sigma-Aldrich, Milano, Italy) and human serum from patients with AAA (sPT). Briefly, the cells were settled in 24-well plates and in parallel on µ-Slides 8 well ^high^ Glass Bottom (N.80807, Ibidi GmbH, Gräfelfing, Germany) coated with Bovine Collagen Type I (N.47257, SERVA Electrophoresis GmbH, Heidelberg, Germany). After reaching about 50% confluence, the cells were treated with medium supplemented either with progressively increased amounts of IL-8 (IL-8 10 ng/mL, IL-8 30 ng/mL, IL-8 50 ng/mL), or sPT (2% sPT twice, then 5% sPT), following the protocol schematized in Supplementary Figure S1. Lad (10 μM or 25 μM) was added to culture 4 h before the end of the stimulation. Thereafter, the cell supernatants were harvested from cells in multiwells, which were rinsed in Dulbecco’s phosphate buffer (PBS) and either fixed in 1% paraformaldehyde in PBS (PFA) for histochemistry assays or frozen for RNA and protein extraction. At the end of stimulation, cells in µ-slides were incubated with MitoTracker CMX-ROS (300 nM, 30 min, 37 °C, N. M7512 Invitrogen, ThermoFisher, Waltham, MA, USA) diluted in warm cell culture medium to label active mitochondria, then fixed with 1% PFA.

### 2.3. Immunofluorescence

Fixed cells were permeabilized with 0.1%Triton X-100 in PBS for 10 min, non-specific binding was blocked by 1% bovine serum albumin in PBS (blocking) for 30 min RT. F-actin was stained by Phalloidin (1:100, 30 min RT, P5282, Sigma-Aldrich, Milano, Italy) diluted in blocking. The cell phenotype was verified by Rabbit anti-Human VE-Cadherin (1:200, o/n 4 °C, AB-J3158, Immunological Sciences, Roma, Italy) or Mouse anti-human α smooth muscle actin (1:500, o/n 4 °C, MAB1420, R&D, Bio-Techne, Minneapolis, MN, USA), revealed by secondary antibody (Donkey-anti-Mouse IgG or Donkey-anti-Rabbit IgG AlexaFluor488-conjugated, diluted 1:500, incubated for 45 min RT, Thermo Fisher Life technologies Inc., Carlsbad, CA, USA). Upon rinse in PBS, the nuclei were stained with 4′,6-diamidino-2-phenylindole (DAPI, 0.2 nmol/L, 10 min, RT, D9542, Sigma-Aldrich, Milano, Italy). Slides mounted within 15%glycerol in PBS were analyzed under Olympus FluoVIEW 3000 RS confocal microscope using a UPLSAPO 60XS (NA 1.3) *Sil* objective. Multiframe images were obtained, then combined in 2D projections by the Fiji open-source software, ImageJ-based. The 2D images were collected in panels by using AdobePhotoshop CS [16].

### 2.4. Gel Zymography

Gelatin zymography was performed to evaluate the MMP gelatinolytic activities into cell supernatants. Briefly, samples diluted in Laemmli buffer were resolved on 8% SDS-PAGE containing 0.1% gelatin. Gels were washed in 2.5% Triton X-100 at RT on orbital shaker, then incubated overnight at 37 °C in 0.05 M Tris-HCl pH 8.8 containing 0.02% NaN_3_ and 5 mMCaCl_2_. Staining with 0.1% Coomassie blue and de-staining with 5% methanol and 7% acetic acid solution followed. Areas of digestion were visualized as non-stained regions. Optical densitometry was performed with Fiji software [16].

### 2.5. NADH/βNADPH Diaphorase Histochemistry

Fixed HAOEC were rinsed in PBS for 5 min at room temperature, then incubated (o/n, 37 °C) in 1 mM NADH/βNADPH and 0.2 mM Nitroblue Tetrazolium (cod. N. 10128023001, 10128024001, and N6876, respectively, Sigma-Aldrich, Milano, Italy) diluted in 100 mM Tris-HCl buffer pH 8.0 containing 0.1% Triton X-100. The oxidation of NAD(P)H generated hydrogen ions which bind to Nitroblue Tetrazolium, allowing the formation of a stable blue formazan. The redox reaction was stopped rinsing cells in PBS, and absorption was quantified by Victor 3V reader (450–630 nm). The cells were also visualized and photographed using an Eclipse 55i microscope equipped with a DS-L1 camera (Nikon, Tokyo, Japan) [17].

### 2.6. ATP Assays

ATP content was determined in cell supernatants by a bioluminescence assay based on luciferase’s absolute requirement for ATP in producing light (ATP Determination Kit, A22066, Invitrogen, ThermoFisher, Waltham, MA, USA) according to the manufacturers’ instruction, as previously described [18]. Samples were run in triplicate and luminescence was measured on Infinite F200 microplate reader (TECAN Group Ltd., Männedorf, Switzerland).

### 2.7. RNA Isolation and Quantitative Real-Time PCR

Total mRNA was extracted using miRNeasy Tissue/Cells Advanced Mini Kit (Qiagen, Hilden, Germany). Synthesis of cDNA from 200 ng of mRNA was carried out by reverse transcription using High-Capacity RNA-to-cDNA Kit (Invitrogen, Carlsbad, CA, USA). Quantitative real-time PCR (RT-qPCR) was performed using TaqMan^®^ Universal Master Mix II and Taqman primer/Fam-labeled probes (Table S1, Applied Biosystems, Foster City, CA, USA) determining the relative levels of the expressed genes. All the procedures were carried out according to the manufacturers’ instruction. Samples were run in triplicate, target gene levels were normalized to that of βactin mRNA, and relative expression was determined using the 2^−ΔCt^ method. The gene expression is presented as ratio applying the formula:

R = 2^−ΔCt^ (stimulated cells)/2^−ΔCt^ (untreated cells).

### 2.8. Statistics

The normality of the distribution of experimental data was assessed by Shapiro–Wilk test. Parametric or non-parametric tests were used depending on the normality of the data distribution. Differences among variables were calculated by using Wilcoxon or *t*-test, and with mixed-effects analysis or Friedman test for multiple comparisons. Simple linear regression or non-linear fits were evaluated to relate experimental parameters. Probability values <0.05 were considered significant. Prism 9.5.0 software (Graphpad Software Inc., La Jolla, San Diego, CA, USA) was used.

## 3. Results

### 3.1. Primary HAOEC Conditioned with IL-8 and Treated with Lad: Gene Expression and MMP9 Activities

**Figure 1:**
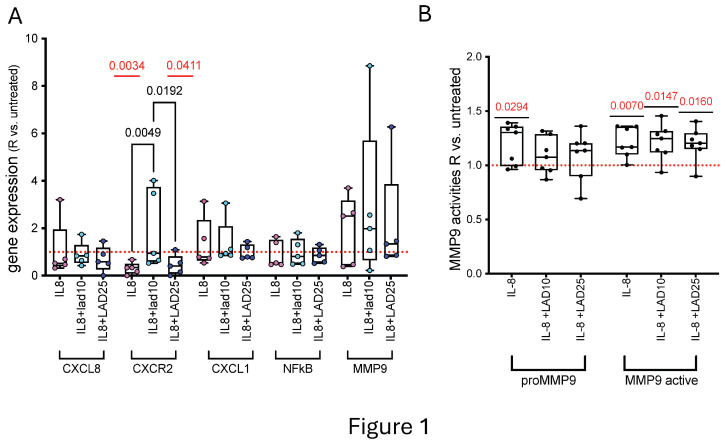
**Gene expression levels and released MMP9 activities in IL-8 conditioned HAOEC.** The expression level of *CXCL8, CXCR2, CXCL1, NFκB*, and *MMP9* genes measured by RT-qPCR in HAOEC (n = 5) is shown (**A**). Changes in MMP9 gelatinolytic activities in cell supernatants (n = 7) are plotted (**B**). Data are presented as ratio vs. untreated cells in boxes (min to max). Dots indicate single-sample values and *p* < 0.05 significant differences among treatments by Friedman test between single treatment and vs. untreated cells by Wilcoxon or *t*-test, depending on the normality of distribution. Pairwise comparisons are shown in each plot in red (comparison vs. untreated cells) and black (comparison between conditioned cells).

To evaluate pro-aneurysmatic effects of chronic, progressively increasing concentrations IL-8 over aortic endothelial cells and the modulatory effects of Lad, we conditioned HAOEC with IL-8 before treating cells with the drug (Figure S1). IL-8 induced downregulation of CXCR2 gene expression with respect to untreated cells, restored by 10 μM Lad but not by 25 μM Lad (Figure 1A).

No significant changes in *CXCL8, CXCL1, NFKB*, and *MMP9* gene expressions were found in the presence/absence of Lad (Figure 1A), thus supporting a close functional relation between IL-8 and its receptor.

Lad antagonism on CXCR2 in AAA-cultured tissue modulated the activities of MMP9 isoforms [7]. To understand whether the modulation of released MMP9 was a downstream effect of IL-8 stimulus and Lad treatment, gel zymography was performed on HAOEC supernatants. Gelatinolytic activities of both proMMP9 and MMP9 active isoforms increased in the supernatants of HAOEC conditioned with IL-8 (Figure 1B and Figure S2A), whilst the activities of proMMP9 by HAOEC treated with 10 μM Lad and 25 μM Lad were assimilable to untreated cells, indicating recovery action. At difference, active MMP9 was significantly higher in the presence of both 10 μM Lad and 25 μM Lad than in untreated HAOEC (Figure 1B). These data supported the role of IL-8 in negative remodeling of the extracellular matrix and the potential, even partial, regulatory efficacy of Lad.

### 3.2. Primary HAOEC Conditioned with IL-8 and Treated with Lad: Energetics and Cytoskeleton

In HAOEC conditioned with IL-8, the NADH and NADPH diaphorases (oxidative activity of mitochondrial complex I and V, respectively) failed to show significant alterations (Figure S2B,C). In agreement, no change in the amounts of the released ATP was found, excepted for a moderate increase in cells conditioned with IL-8 and treated with 10 μM Lad vs. those only stimulated with IL-8 (Figure S2B). Interestingly, MitoTracker showed that the intracytoplasmic mitochondria organization changed in HAOEC treated with IL-8, with condensation of active mitochondria in the perinuclear area, and partially recovered upon treatment with Lad (Figure S2D). A slight alteration in VE-Cadherin pattern and the formation of stress fibers confirming the responsiveness of HAOEC to IL-8 and a structural recovery with Lad treatment were observed (Figure S2E,F).

### 3.3. Primary HAOEC Conditioned with Human Serum and Treated with Ladarixin: Gene Expression and MMP9 Activities

To assess whether and how the pro-aneurysmatic response of HAOEC changes in the presence of complex inflammation-related stimulation including IL-8, the cells were conditioned with serum from patients with AAA (sPT) (Figures S1 and S3A).

**Figure 2:**
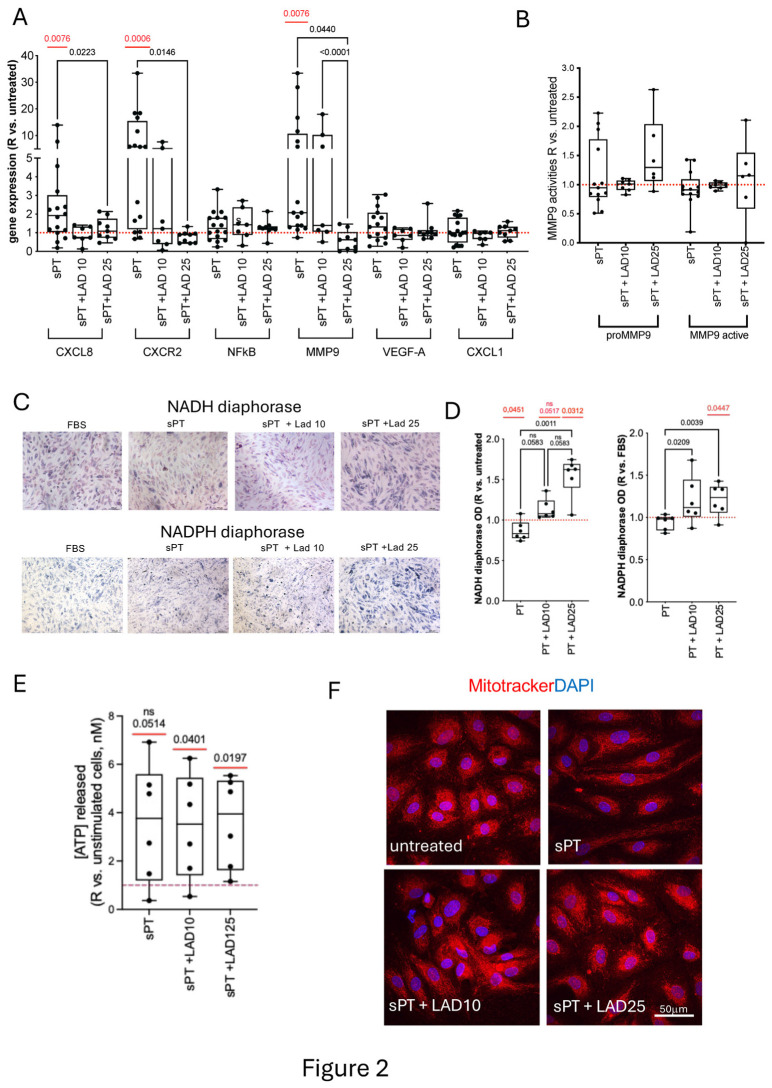
**Gene expression levels, MMP9 gelatinolysis, NAD(P)H diaphorases, and ATP release in sPT-conditioned HAOEC.** Gene expression by RT-qPCR of CXCL8, CXCR2, NFκB, MMP9, VEGF-A, and CXCL1 after cells conditioning +/- treatment with Lad is presented (n = 16, n = 7, and n = 9, respectively, (**A**)). Changes in gelatinolysis by released MMP9 after cells conditioning +/- treatment with Lad (n = 7) are presented (**B**). Representative light microscopy images of NADH and NADPH diaphorase histochemistry (objective 10x) are shown (**C**). Quantification of NADH/NADPH diaphorase activities (n = 6) (**D**) and ATP release in cell supernatants (n = 6, (**E**)) are plotted. Representative confocal microscopy images of MitoTracker-labeled mitochondria (red, (**F**)) are shown on HAOEC. Nuclei are stained with DAPI (blue). Data in (**A**,**B**,**D**,**E**) are presented as ratio vs. untreated cells in boxes (min to max). Dots indicate single-sample values and *p* < 0.05 significant differences by mixed-effects analysis in (**A**,**B**). Pairwise comparisons are shown in each plot in red (comparison vs. untreated cells) and black (comparison between conditioned cells).

Upregulation of CXCL8, CXCR2, and MMP9 genes without changes in NFkB, VEGF-A, and CXCL1 (Figure 2A) were found in HAOEC conditioned with sPT. Treatment with 25 μM Lad reverted the upregulated gene expression to untreated cell level.

Despite the increase of *MMP9* expressed gene, no changes in the gelatinolytic activities of released MMP9 protein were detected in HAOEC conditioned with sPT with respect to untreated cells, either in the presence/absence of Lad (Figure 2B and Figure S3B), suggesting a regulation of metalloproteinases not restrained to IL-8 action or requiring high cytokine levels. In HAOEC conditioned with sPT in the presence of 25 μM Lad, the gelatinolysis by MMP9 active was associated to that of proMMP9 and to that in the absence of Lad (Figure S3C). The gelatinolysis by proMMP9 was linearly related to the amount of CXCL1 and IL-1β proteins in sPT (Figure S3D); this association reverted with 10 μM Lad but not with 25 μM Lad. Moreover, in the presence of 10 μM Lad, both MMP9 activities resulted negatively related also to IL-8 and IL-1βcontent in sPT.

### 3.4. Primary HAOEC Conditioned with Human Serum and Treated with Lad: Energetics and Cytoskeleton

In HAOEC conditioned with sPT, the NADH diaphorase was negatively related to the amount of IL-8 in sPT and positively to that of IL-1β (Figure S4A). The sPT-conditioning led to a decrease in NADH diaphorase, consistent with oxidative stress. Recovery was observed in the presence of Lad, significant with 25 µM Lad, which was also higher than untreated cells (Figure 2C,D). This was supportive of oxidoreductase regulation by Lad. Conversely, the NAPDH diaphorase did not change in HAOEC conditioned with sPT vs. untreated but was significantly lower than in cells treated with 10 μM Lad and 25 μM Lad (Figure 2C,D). The NADH and NADPH diaphorase activities were both higher in 25 μM Lad-treated HAOEC vs. untreated. Analogously, the release of ATP did not change following sPT conditioning, but increased with respect to untreated cells in HAOEC treated with both 10 μM Lad and 25 μM Lad (Figure 2E). Taken together, these data are compatible with an effect of increasing mitochondrial activity by Lad in sPT-conditioned cells.

At difference from what observed with IL-8, the mitochondria appeared diffusely distributed following sPT. A perinuclear localization comparable to that of untreated cells was observed with 25 μM Lad (Figure 2F). The actin cytoskeleton was altered with formation of peripheral stress fibers by conditioning with sPT, and a minor rescue of thin fibers was observed only with 25 μM Lad (Figure S4B).

### 3.5. Primary HAOSMC: Conditioned with IL-8 and Treated with Lad

**Figure 3:**
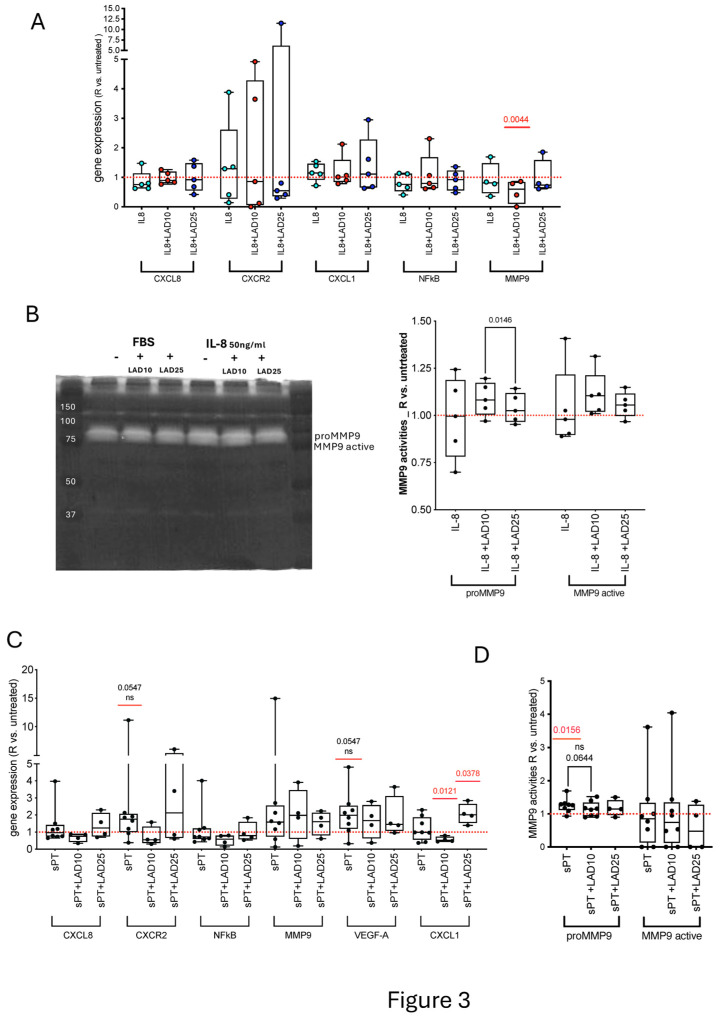
**Gene expression levels and MMP9 activities in IL-8 and sPT-conditioned HAOSMC.** Gene expression by RT-qPCR of *CXCL8, CXCR2, NFκB, MMP9, VEGF-A*, and *CXCL1* after HAOSMC conditioning with IL-8 +/− treatment with Lad (n = 5, (**A**)). Representative gel zymography image of HAOSMC conditioned with IL8 and changes in MMP9 gelatinolysis (n = 5, (**B**)) are presented as ratio vs. untreated cells. Gene expression of *CXCL8, CXCR2, NFκB, MMP9, VEGF-A,* and *CXCL1*, by RT-qPCR in sPT-conditioned HAOSMC +/- treatment with Lad (n = 8, n = 4 and n = 4, respectively, (**C**)) and changes in MMP9 gelatinolysis (n = 5, (**D**)) are shown as ratio vs. untreated cells. Data are presented as boxes (min to max). Dots indicate single-sample values and *p* < 0.05 significant differences among treatments by mixed-effects analysis, between single treatment and untreated cells by Wilcoxon or *t*-test depending on the normality of distribution. Pairwise comparisons are shown in each plot in red (comparison vs. untreated cells) and black (comparison between conditioned cells).

In HAOSMC conditioned with IL-8, no significant change in the expression of CXCL8, CXCR2, CXCL1, NFKB, and MMP9 genes was determined. Treatment with 10 μM Lad, but not with 25 μM Lad, downregulated the MMP9 gene in IL-8-conditioned HAOSMC compared to untreated cells (Figure 3A).

The zymography on supernatants from HAOSMC conditioned with IL-8 failed to show changes in MMP9 protein activities with respect to untreated cells (Figure 3B). Moreover, although a significant difference was found in proMMP9 gelatinolysis between IL-8-conditioned HAOSMC treated with 10 μM Lad vs. Lad 25 µM, there was no difference vs. untreated cells. Mitotracker and α smooth muscle actin indicated that no dramatic changes were also induced at mitochondrial (Figure S5A) and cytoskeletal levels (Figure S5B). These data supported a non-prominent role for IL-8 pathway in HAOSMC, unaffected by Lad.

### 3.6. Primary HAOSMC Conditioned with Human Serum and Treated with Ladarixin

No significant modulation of gene expression was measured upon sPT-conditioning of HAOSMC, but trends towards upregulation of CXCR2 and VEGF-A were observed (Figure 3C). Moreover, an opposite modulation of CXCL1 with 10 μM Lad (downregulation) and 25 μM Lad (upregulation) in conditioned vs. untreated HAOSMC was found, suggestive of a rebound effect with Lad 25 µM.

The proMMP9 gelatinolytic activity significantly increased in HAOSMC conditioned with sPT vs. untreated cells, and a trend towards reduction was observed following treatment with 10 μM Lad (Figure 3D and Figure S5C). The data were not related to the amounts of inflammatory mediators in sPT. Taken together, these findings support a scarce responsiveness of HAOSMC to sPT and a limited reactivity to Lad in non-activated conditions.

## 4. Discussion

Since the early 90s, AAA development has been causally related to increase of IL-8 cytokine amount and activity at aortic wall level [6,19,20,21]. A functional link between CXCR2/CXCL1/IL-8 axis and MMP9 activity in cultured lesioned aortic tissues from AAA and the MMP9 modulation via IL-8 receptor antagonism was demonstrated [7]. We show differences in the responsiveness of primary aortic cells to IL-8 and to serum from patients with AAA, and the modulatory effects over cell responses by the IL-8 receptor antagonist Lad, in the optics of viable therapeutic approaches against aortic damage.

IL-8 promoted in dose-dependent manner, either survival (via MMP9 enhancement and capillary tube formation) or dysfunction (via alteration of cytoskeleton and tight junctions) of *in vitro* endothelial cells [22,23], and was a functional modulator of SMC [24,25]. The decrease of *CXCR2* upon exposure of HAOEC to mature IL-8 (under conditions of progressively increasing, chronic inflammatory stimulation [26]) may represent a defensive reaction against alteration of the endothelial chemoattractant and of cell-to-cell interaction properties, compatible with first-line localization and protective role of HAOEC *in vivo* [27]. The absence of changes in other genes may indicate no activation of autocrine pathway or angiogenesis [28]. Instead, the IL-8-associated increase of the MMP9 protein activity is compatible with the chemokine role as extracellular matrix degradation enhancer. None of these effects is elicited in HAOSMC, thus indicating different intracellular mechanisms associated with IL-8 processing by vascular cells. Biological activities of IL-8 change upon site-specific proteolytic modification, mediated by MMP9 and other metalloproteinases, whose deregulation in pathological conditions like AAA supports the relevance of IL-8 cleavage in inflammatory settings [29].

Differences in responsiveness to IL-8 may be related to cell diversity in the vascular beds. Human saphenous vein cells provide an example: isolated SMC were able to produce and secrete IL-8 but lacked binding sites, whilst endothelial cells produced and bound IL-8 [30]. The aortic artery cells differ from those of the saphenous vein, as HAOSMC endogenously express higher levels of *CXCL8* and *CXCR2* than HAOEC but scarcely react to the prolonged conditioning with IL-8.

The behavior of HAOEC is distinct from that of HAOSMC also with sPT-conditioning. This complex inflammatory stimulus triggers early signs of endothelial dysfunction in HAOEC (i.e., upregulation of *CXCL8, CXCR2*, and *MMP9* genes in the presence of variable MMP9 protein activity) but extracellular matrix/angiogenesis-related changes in HAOSMC. (i.e., increased proMMP9 gelatinolysis and borderline significant upregulation of *CXCR2* and *VEGF-A* genes). Despite the intrinsic constitutive differences, both HAOEC and HAOSMC respond to pharmacological antagonism of IL-8 receptors by Lad, which demonstrates rescuing efficacy.

In HAOEC, the sPT effect over MMP9 protein is linearly related to the serum content of CXCL1 (member of the ELR CXC family, as IL-8 [31,32]) and to IL-1β. Conversely, the efficacy of 10 μM Lad in rescuing gelatinolysis is negatively associated to the amounts of IL-8, IL-1β, and CXCL1 in sPT [7], and the rebound effect on proMMP9 by 25 μM Lad to the content of CXCL1. These findings highlight a non-exclusive role of IL-8 in the pro-aneurysmatic activation of aortic endothelium and suggest a connection at endothelial level between the drug action and the multifactorial composition of the stimulus.

Interplay between Lad and CXCL1 is also found in HAOSMC conditioned with sPT, where 10 μM Lad and 25 μM Lad exert opposite actions over gene expression, assimilable to stabilization and rebound effects, respectively. The association of CXCL1 increase to the inflammatory activation of rat vascular SMC induced by hypertension [33], to the causes of hypertension and MMP9 release in human diseases [32,34], and to AAA growth [35] support the potential relevance of Lad effects over CXCL1 in the perspective of AAA therapy. Together with the higher efficiency of 10 μM Lad vs. 25 μM Lad in modulating proMMP9 gelatinolysis in aortic cells, and in agreement with previous data on AAA tissue cultures [7], these findings suggest 10 μM Lad as a candidate dose for further studies.

Consequences of inflammation of the vascular wall and of intracellular pathways activated by IL-8 [36] may be multifaceted, and causal relationships with SMC remodeling and oxidative stress/respiration were demonstrated, pointing to a role for NADPH oxidases [3,37]. Although IL-8-driven alteration of NAD(P)H-dependent redox balance and ATP production at endothelial level [38] was reported in sepsis [39], if and how inflammation triggers endothelial dysfunction via cell remodeling and redox activity alteration under AAA-like conditions is almost unexplored. Our findings show that IL-8 leads to reinforcement of F-actin peripheral fibers [23] and mitochondria aggregation in HAOEC but not in HAOSMC. These changes are not paralleled by variation in NAD(P)H diaphorases (related to respiratory chain activity and nitric oxide synthesis, respectively [11,13,40]), and in ATP release. In sPT-conditioned HAOEC, the NADH diaphorase is associated to the sPT content of IL-8 and of IL-1β in opposite ways, and conditioning induces a decrease in both diaphorase activities, a trend towards increasing ATP secretion, and mitochondria spatial reorganization. Overall, these findings support early-stage remodeling in HAOEC, insufficiency of IL-8 but need of multiple mediators to trigger bioenergetic changes.

As in whole vessels ATP fuels vasodilation and maintenance of vascular tone [41], the variation in ATP released by HAOEC in the absence of shear stress/blood flow but presence of inflammatory stimuli may provide preliminary indication of how inflammation contributes to alter the artery diameter in AAA. Further studies investigating this mechanism under either static or dynamic conditions are required.

In the sPT-conditioning setting, treatment with Lad has augmentative, dose-dependent effects over energetics in HAOEC, and 25 μM Lad restores the mitochondrial pattern, significantly increasing diaphorases and ATP release. The reaction of HAOEC to Lad extends the drug effects beyond extracellular matrix regulation, possibly to the reversion of the energetic unbalance due to sPT, results in mitochondria overwork buffered by ATP release, and underlines that drug calibration is pivotal.

The pattern of aortic cell responsiveness to Lad is also supportive of the need for combination therapy in AAA, in agreement with the failure in patients of therapies based on IL-8 inhibition, and effective on animal models [36].

## 5. Conclusions

In conclusion, IL-8 chronic stimulus plays a role in the post-translational regulation of MMP9 and in the modulation of *CXCR2* gene in HAOEC, and AAA patient’ serum acts more widely over expressed genes of the CXCR2/CXCL1/IL-8 axis, underlining the synergistic/antagonistic interplay among serum inflammatory mediators in affecting the vascular wall. Moreover, the action of sPT, but not of IL-8, on HAOEC involves unbalance of redox-associated NAD(P)H enzymes, ultimately affecting the release of ATP.

HAOSMC are almost unresponsive to IL-8 but respond to AAA patient’ serum at MMP9 post-translational level, compatibly with the prominent increase of extracellular matrix degradation in AAA.

IL-8 antagonism by Lad is partially efficient in recovering the IL-8 effects and fully efficient against those due to sPT. Characterizing whether Lad action in aortic cells involves specific engagements other than with IL-8 receptors goes beyond the purposes of this study. Finally, rebound effects following 25 μM Lad suggest being careful with dose calibration and indicate 10 μM Lad as a possible therapeutic candidate for vascular damage in AAA, warranting further studies.

## Data Availability

The main data presented in this study are available either in the article tables or in Supplementary Materials. Data from serum donors are subjected to privacy law and available on request from the corresponding author.

## Supplementary Materials

The following supporting information can be downloaded at: https://www.mdpi.com/article/10.3390/cells14211713/s1, Figure S1 Schematic representation of the cell treatments. Table S1. 1 FAM-probes for RT-qPCR. Table S2. Basic characteristics of blood donors. Figure S2 HAOEC conditioned with IL-8. Figure S3. HAOEC conditioned with sPT. Figure S4. HAOEC conditioned with sPT. Figure S5. MMP9 activities, staining Mitotracker and αSMA of conditioned HAOSMC.
