# Supplementary material for: Ladarixin Potential over the Effects of IL-8 and of Serum from Patients with Abdominal Aortic Aneurysm on Human Aortic Cells
Source: Cells. 2025 Oct 31;14(21):1713. doi: 10.3390/cells14211713 (PMC12610885; doi:10.3390/cells14211713)
Supplement: Supplementary file 1 [file cells-14-01713-s001.zip › cells-3925270-supplementary.pdf]

CELL CULTURE

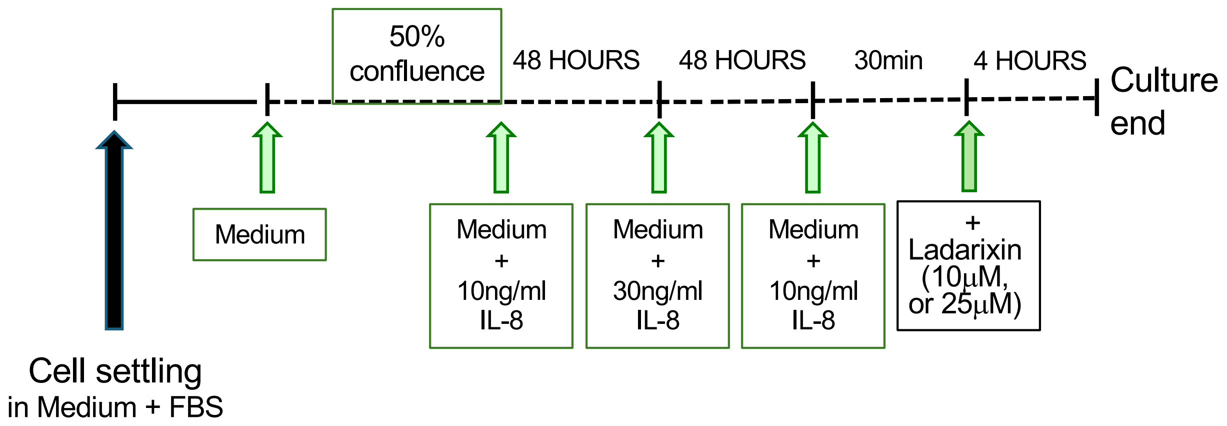

CELL CULTURE

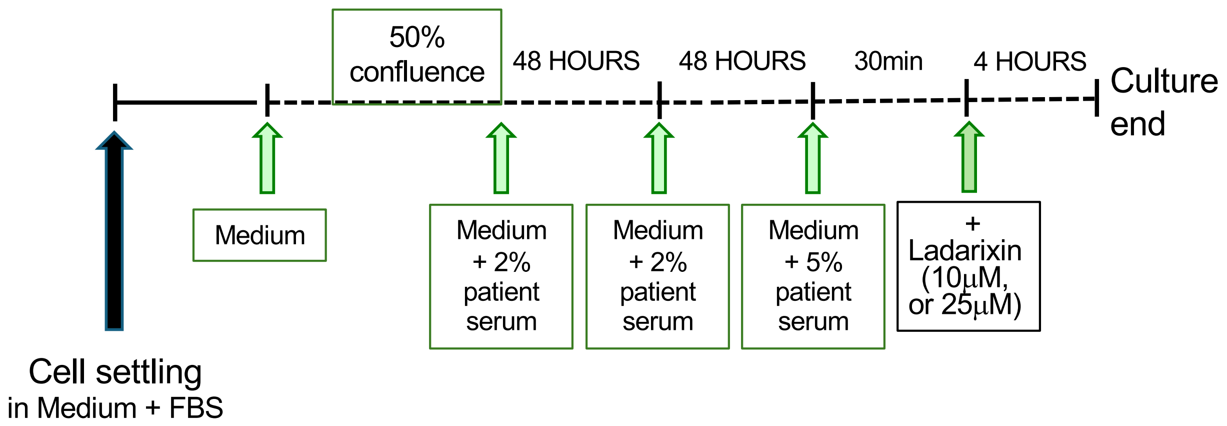

Figure S1 Schematic representation of the cell treatments

Table S1. FAM-probes for RT-qPCR

Name	Assay ID
ACTB	Hs01060665_g1
CXCL1	Hs00236937_m1
CXCL8	Hs00174103_m1
CXCR2	Hs01891184_s1
MMP9	Hs00234579_m1
NFKB1	Hs00765730_m1
VEGF-A	Hs00900055_m1

Supplementary TABLE S2. Basic characteristics of blood donors

demographic & clinical data of patients with AAA (n=14)

age	(mean_years, SD)	70 ± 7
smoking status	not smoker [n (%)]	6 (43)
	ex- smoker [n (%)]	6 (43)
	active smoker [n (%)]	2 (14)
hypertension	[n (%)]	11 (79)
dyislipidemia	[n (%)]	7 (50)
type II diabetes	[n (%)]	2 (14)
AAA max diameter	[mean± SD(mm)]	58 ±14
hemodinamically significant carotid atherosclerosis	[n (%)]	5 (36)
periphery artery disease	[n (%)]	8 (57)
previous coronary STENT/PTCA	[n (%)]	2 (14)
coronry artery disease	[n (%)]	3 (21)
pregressed myocardial infarction	[n (%)]	0
atrial fibrillation	[n (%)]	1 (7)
chronic kidney disease	[n (%)]	1 (7)
others	[n (%)]	11 (71)

hematologic data at hospitalization

parameter (range)	*mean ± SD*
WBC (4 ,8 - 10 ,8)	8,2 ± 2,6
RBC (4 ,7 - 6 ,1) *	4,5 ± 0,8
Hg (14 - 18)*	13,2 ± 2,3
hematocrit (42 - 52)*	40,2 ± 6,1
MCV (80 - 94)*	89,8 ± 8,1
MCH (27 - 34)	30,2 ± 3,3
RDW (11 ,5 - 14 ,59)	14,2 ± 1,9
PLT (130 - 400)	196 ± 51
MPV (9 ,1 - 12 ,5)	10,9 ± 0,6
eosinophils (0 - 0 ,45)	0,1 ± 0,1
basophils (0 - 2)	0,1 ± 0,1
neutrophils (1 ,8 - 7 ,7)	5,7 ± 2,6
linfocytes (1 - 4 ,8)	1,6 ± 0,7
monocytes (0 ,2 - 0 ,8)	0,6 ± 0,1
glucose (60 - 100)	115 ± 22
urea (10 - 50)	42 ± 20
Sodium (135 - 148)	140,7 ± 2,3
Potassium (3 ,5 - 5)	4,3 ± 0,5
Calcium (2 ,1 - 2 ,6)	2,20 ± 0,14
amilase (28 - 100)	76 ± 34
creatinine (0 ,5 - 1 ,25)	1,28 ± 0,75
PT (sec)	14,9 ± 1,5
INR	1,15 ± 0,12
APTT (sec)	30,4 ± 3,0

Legend: WBC, white blood cell; RBC red blood cells; Hg, hemoglobin; Mean corpuscular volume, MCV; Mean corpuscular hemoglobin, MCH; RBC distribution width, RDW; C-reactive protein , CRP; prothrombin time, PT;International Normalized Ratio for PT, INR; Activated Partial Thromboplastin Clotting Time, APTT.

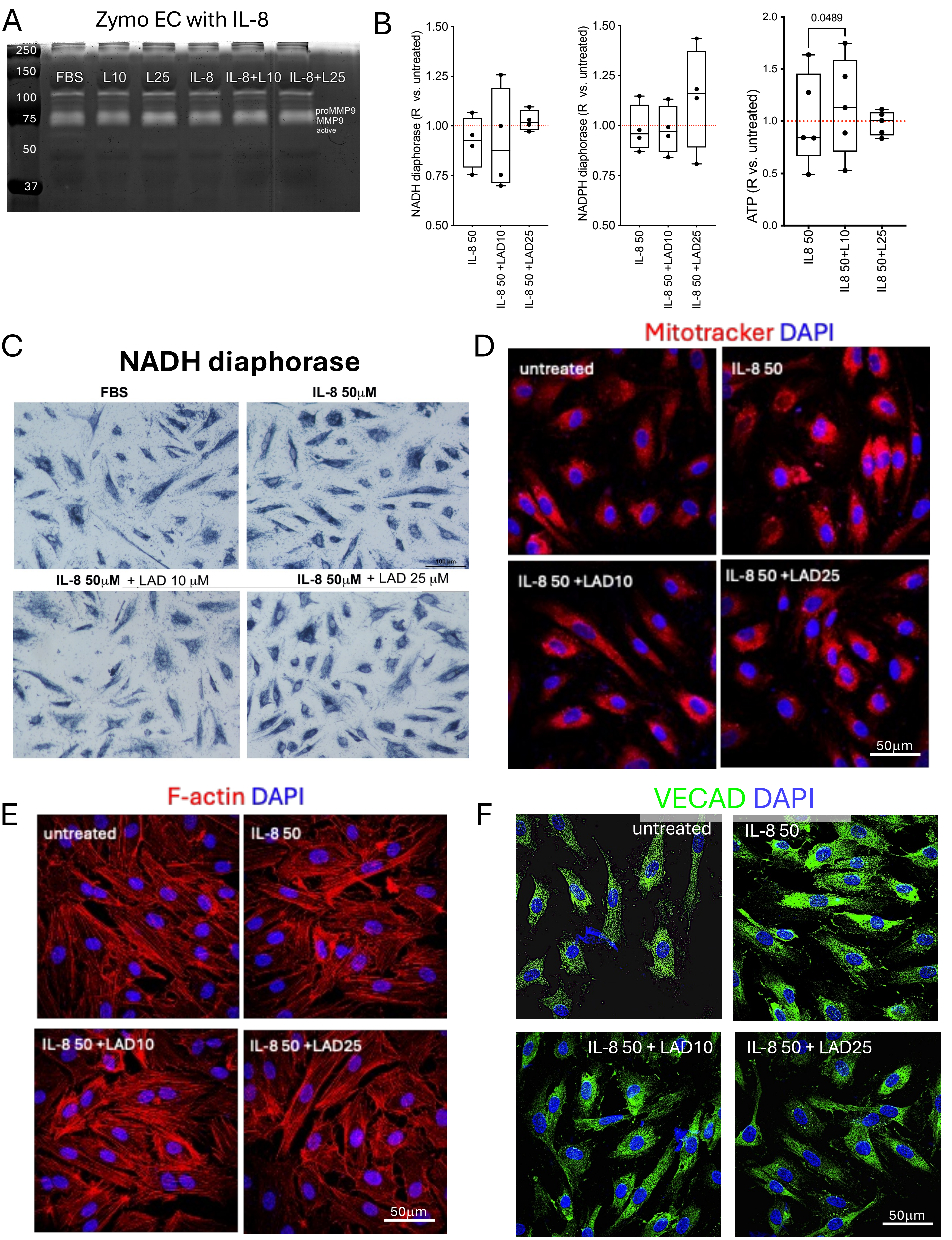

Figure S2 HAOEC conditioned with IL-8 Representative gel zymography image of HAOEC with IL-8 +/- treatment with Lad is shown (**A**). NADH and NADPH diaphorase activities of HAOEC with IL-8 +/- treatment with Lad and quantification in cell supernatants of released ATP (n= 4, n=4 and n=5, respectively) are plotted (**B**). Dots indicate single sample values and $p<0.05$ significant difference by t-test. Representative images of NADH diaphorase histochemistry (**C**), confocal microscopy images of MitoTracker-labelled mitochondria (red, **D**), of Phalloidin -labelled F-actin cytoskeleton (red, **E**) and of VE- Cadherin (green, **F**) are shown on HAOEC. Nuclei are stained with DAPI (blue).

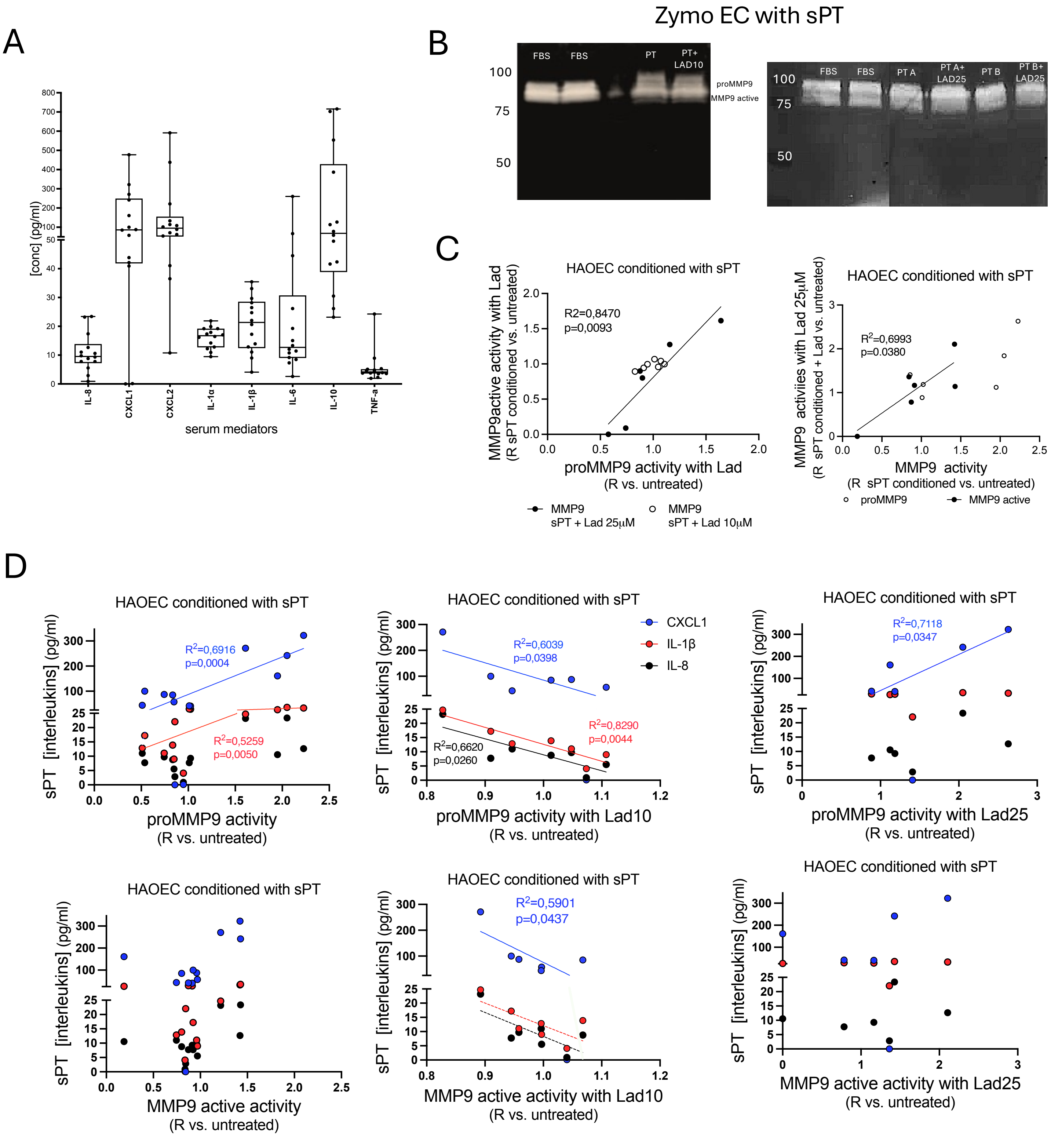

Figure S3. HAOEC conditioned with sPT. Quantification of selected. mediators in the patient serum used for cell conditioning (A). Representative gel zymography image of HAOEC with IL-8 +/- treatment with Lad is shown (**B**). Linear correlation between the activity of MMP9 active and that of proMMP9 in sPT - conditioned HAOEC treated with Lad (**C**). Correlations between CXCL1, IL-1β and IL-8 concentrations in sPT and MMP9 activities are shown (D).

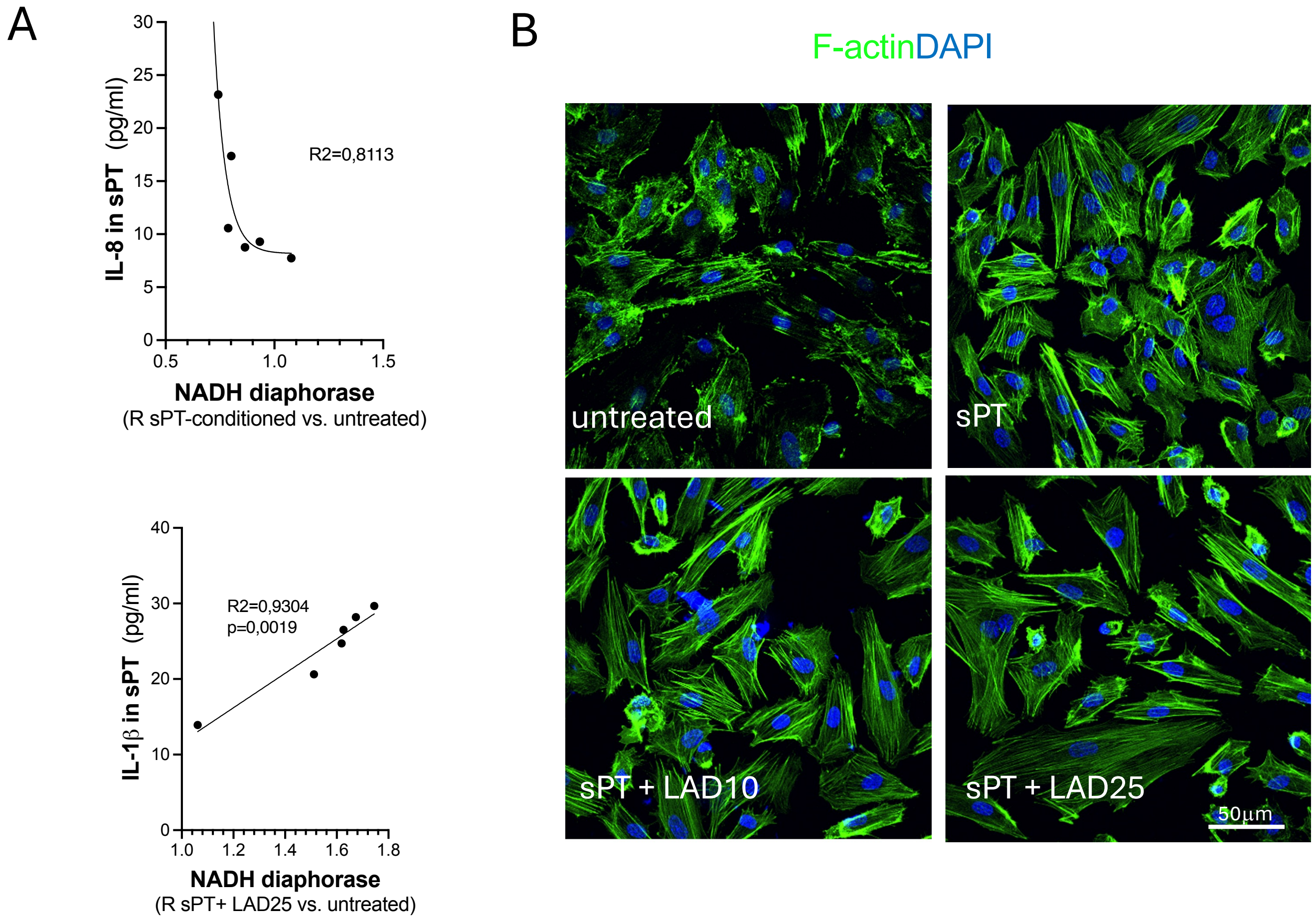

Figure S4. HAOEC conditioned with sPT. Relationships between NADH diaphorase activity and the concentration of IL-8, IL-1β in sPT, respectively, are shown (**A**). Representative confocal microscopy images of MitoTracker-labelled mitochondria **(**red, **B)** and Phalloidin - labelled F-actin cytoskeleton (green, **C)** are shown on HAOEC. Nuclei are stained with DAPI (blue).

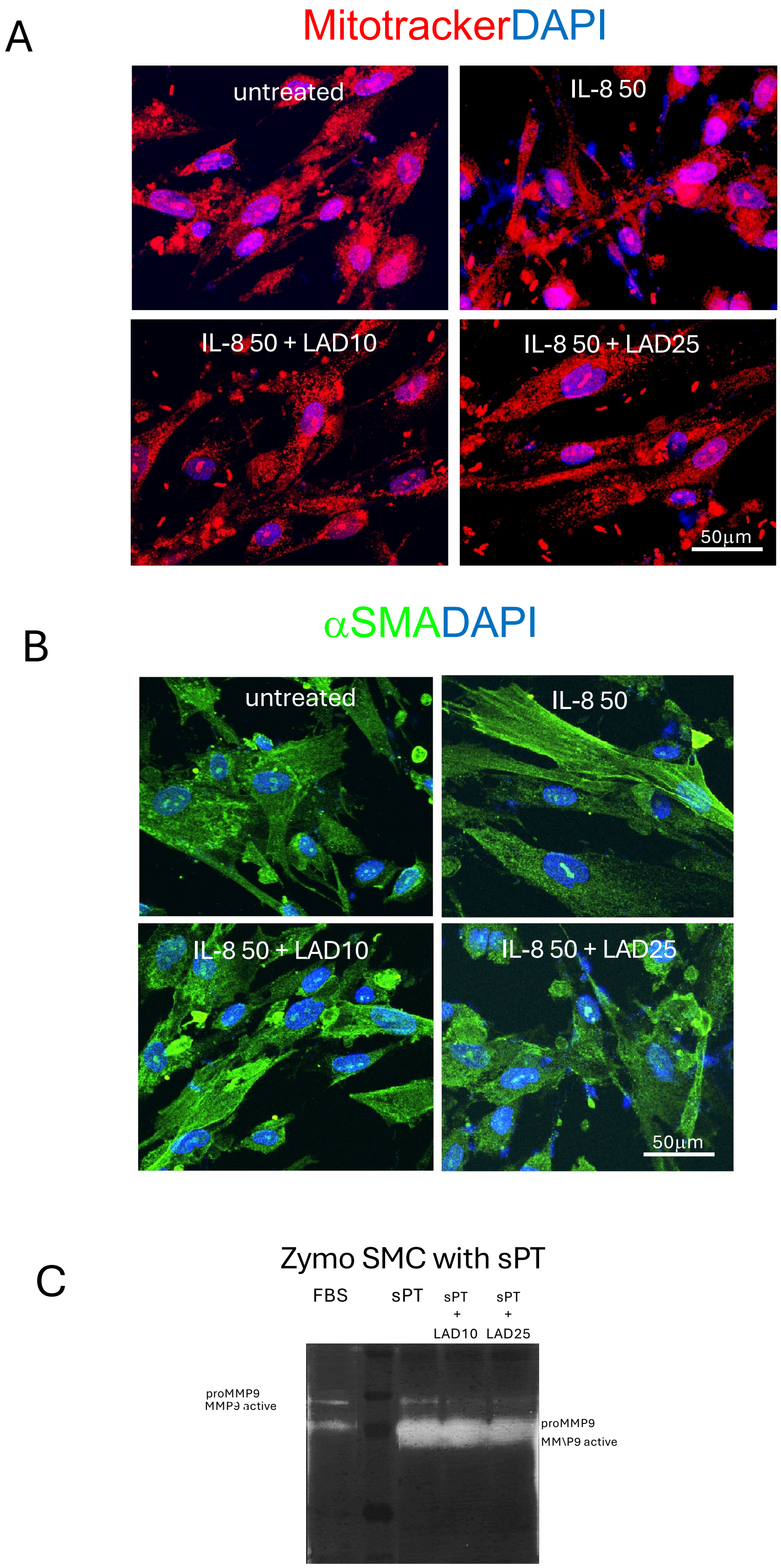

Figure S5. MMP9 activities, staining Mitotracker and αSMA of conditioned HAOSMC. Representative confocal microscopy images of MitoTracker-labelled mitochondria **(**red, **A)** αSMA **(**green, **B)** are presented on HAOSMC. Nuclei are stained with DAPI (blue). Representative gel zymography image of HAOSMC conditioned with sPT **(C).**
